# Mapping Trends in Moyamoya Angiopathy Research: A 10-Year Bibliometric and Visualization-Based Analyses of the Web of Science Core Collection (WoSCC)

**DOI:** 10.3389/fneur.2021.637310

**Published:** 2021-03-02

**Authors:** Danyang Chen, Ge Zhang, Jiahui Wang, Shiling Chen, Jingxuan Wang, Hao Nie, Zhouping Tang

**Affiliations:** ^1^Department of Neurology, Tongji Hospital, Tongji Medical College, Huazhong University of Science and Technology, Wuhan Hubei, China; ^2^Department of Geriatrics, Tongji Hospital, Tongji Medical College, Huazhong University of Science and Technology, Wuhan, China

**Keywords:** bibliometrics, CiteSpace, emerging topics, visualization, HistCite, VOSviewer, moyamoya, Web of Science

## Abstract

**Background:** Moyamoya angiopathy (MMA), which includes moyamoya disease (MMD) and moyamoya syndrome (MMS), is an uncommon cerebrovascular condition characterized by recurrent stroke. We carried out a bibliometric analysis to examine the development of and research trends in MMA research.

**Methods:** Studies published between 2010 and 2019 on MMA were retrieved from the Web of Science Core Collection (WoSCC) on August 14, 2020, and bibliometric and visualization-based analyses were performed by using three different scientometric tools: HistCite, VOSviewer, and CiteSpace.

**Results:** A total of 1,896 publications published in 384 journals by 6,744 authors, 1,641 institutions and 56 countries/regions were included in the analyses. Annual publication outputs increased from 2010 to 2019. The USA, Japan and China were three key contributors to this study field. Capital Medical University, Seoul National University, and Stanford University were three major institutions with larger numbers of publications. Zhang D, World Neurosurgery, Kuroda S, and STROKE were the most prolific author, prolific journal, top co-cited author and top co-cited journal, respectively. The top five keywords during this period were moyamoya disease, revascularization, stroke, children and surgery, while revascularization surgery and RNF213 were the most common frontier topics.

**Conclusions:** In this study, the research trends of global scientific research on MMA over the past decade were systematically analyzed. The study can provide guidance for scholars who want to understand current trends in research in this area and new research frontiers.

## Introduction

Moyamoya angiopathy (MMA) is an infrequent, chronic, and disabling cerebrovascular condition. Clinical features include on-going stenosis and occlusion of the distal part of internal carotid arteries and the proliferation of moyamoya-associated collaterals (1, 2). MMA can be divided into moyamoya disease (MMD) and moyamoya syndrome (MMS). The specific pathological mechanism of MMA remains unclear (3). Revascularization surgery is an effective MMA treatment (2).

In the field of bibliometrics, quantitative and visualization-based analyses were performed on scientific publication data and citation data (4, 5). Through the quantitative analysis of publications available in a library, the research trends in a specific field can be investigated. The Web of Science Core Collection (WoSCC) is the most commonly used database in bibliometric studies (6). Scientometric applications used in bibliometric studies include HistCite (7), VOSviewer (8), and CiteSpace (9). For the past few years, bibliometric analysis has been applied in various biomedical fields (10–12).

As far as we know, no published bibliometric study has focused on MMA research. Here, we visually analyzed the development of and trends in MMA research from 2010 to 2019 using HistCite, VOSviewer, and CiteSpace.

## Materials and Methods

### Data Source and Information Retrieval Strategy

**Figure 1:**
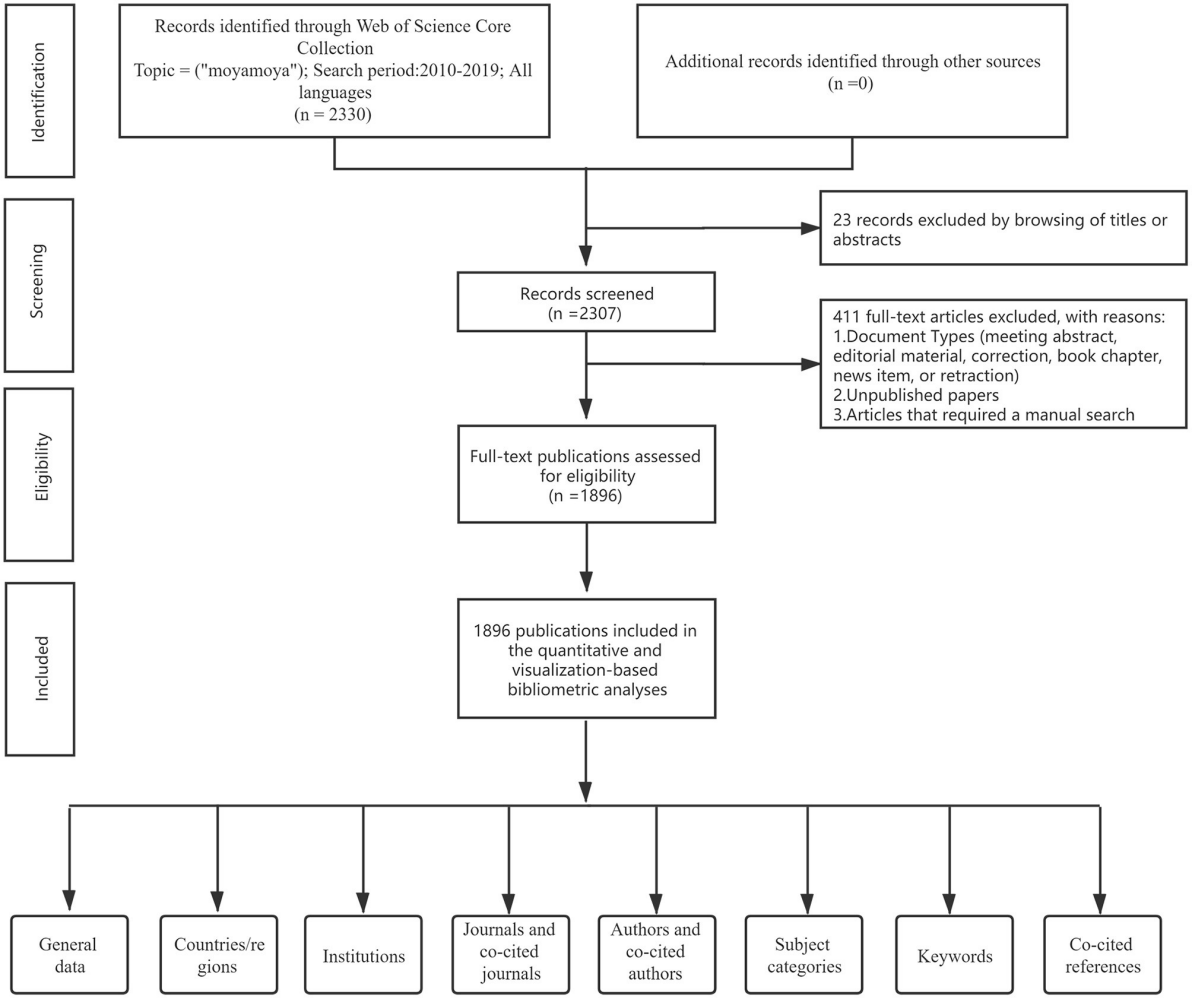
A frame flow diagram. The diagram showed detailed search strategies and selection criteria for MMA publications from WoSCC database and the steps of bibliometric analysis.

A literature search was carried out by using the WoSCC database to collect publications on MMA. All data obtained were appropriate for the bibliometric analysis. All searches were performed under identical conditions to minimize the bias on August 14, 2020. The search strategy was unanimously agreed on by all members of our research team. The advanced search was conducted using the following formula: TS = (“moyamoya”). The detailed search processes and analysis procedures were shown in Figure 1 (13).

### Inclusion Criteria

(a) Peer-Reviewed Articles on MMA
(b) Documents types: articles, reviews, letters, and proceeding papers
(c) Year of publication: 2010–2019
(d) Language type: all languages
(e) Database: Web of Science Core Collection (WoSCC).

### Exclusion Criteria

(a) Articles that required a manual search
(b) Unpublished papers.

### Statistical and Analytical Methods

The full records and cited references of identified publications were exported as plain text and tab-delimited (Win, UTF-8) files for bibliometric analysis and visualization. HistCite (Clarivate Analytics, Philadelphia, PA, USA), CiteSpace 5.3.R4 (Drexel University, Philadelphia, USA) and VOSviewer 1.6.14 (Leiden University, Leiden, Netherlands) were used for data analysis. HistCite is a program used to quickly summarize and analyse publications. In this study, HistCite was applied to confirm annual output, total number of citations, language type and document type. VOSviewer was employed to construct visual maps and summarize prolific countries/regions, institutions, journals, and authors, as well as the top co-cited journals, authors, keywords and references. For VOSviewer, the full counting method was used, and the minimum threshold of the data selected to construct the visual maps depended on specific items such as the institution or journal. CiteSpace, which runs in a Java environment, was used to construct the category map and detect the burst terms of keywords and co-cited references. The parameters used with CiteSpace were set as follows: time slice (2010–2019 by year), text processing (term source: all selection), term type (burst terms), node type (set based on the item), links (strength: cosine; scope: within slices), selection criteria (top 50 objects), and pruning (pathfinder and pruning sliced networks). The linear forecasting model can be described as *f* (*x*) = *ax* + *b*, where *x* is the publication year and *f* (*x*) is the number of publications. The linear fit of the annual number of publications by year was graphed using GraphPad Prism 8 (GraphPad Software, San Diego, CA, USA), and the output was predicted for 2020. Pearson's correlation analysis of the year and annual output was conducted using IBM SPSS Statistics 25.0 software (SPSS Inc., Chicago, USA). Journal impact factors (IFs) were identified according to the 2019 Journal Citation Reports (JCR) released by Clarivate Analytics on June 29, 2020.

A review of ethics was unnecessary because all original data used in this study were from a public database.

## Results

### General Publication Data and the Upward Trend of Annual Output

A total of 1,896 studies published from 2010 to 2019 were used for our bibliometric data analysis. The studies were published in 384 journals by 6,744 authors, 1,641 institutions and 56 countries/regions. There were 20,050 citations in total. The article was the primary document type (*n* = 1,552, 81.86%), followed by the review (*n* = 208, 10.97%), letter (*n* = 132, 6.96%), and proceedings paper (*n* = 4, 0.21%). Languages included English (*n* = 1,873, 98.79%), French (*n* = 10, 0.53%), German (*n* = 7, 0.37%), and Spanish (*n* = 6, 0.32%). Three Eastern Asian languages Japanese, Korean and Chinese, were not included in the database.

**Figure 2:**
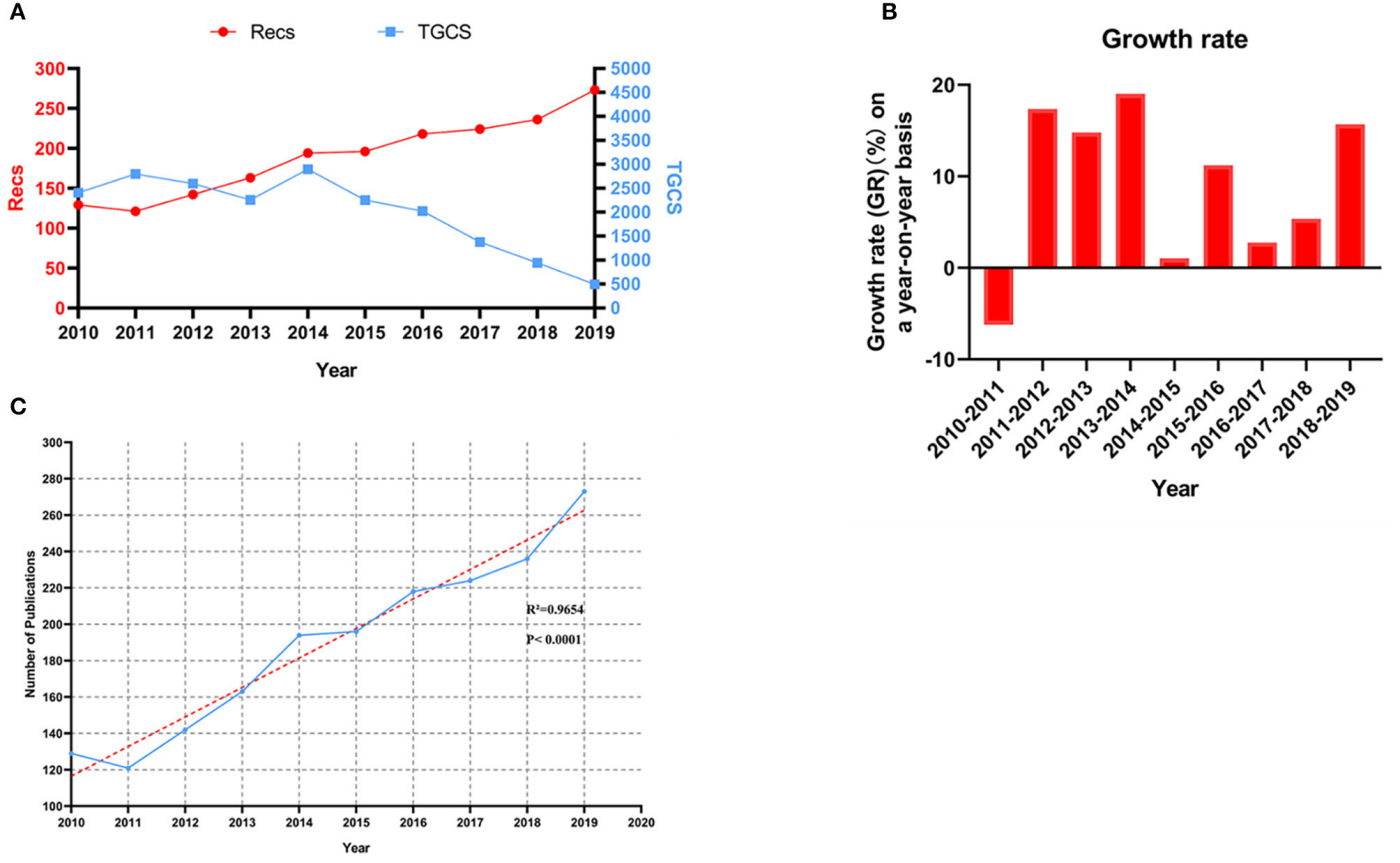
**(A,B)** The annual output, citations and growth rate on a year-on-year basis with regard to MMA during 2010–2019. **(C)** The linear model of the upward trend in the field of MMA (*R*^2^ = 0.9654, *P* < 0.0001). Recs: Number of Records, namely the number of publications for a given year; TGCS: Total Global Citation Score, namely the total count of citations.

It was suggested that the annual output varied by years, and there was a slight increase in output over the last 10 years (Figure 2A). The most prolific year was 2019, in which 273 papers were published, and the minimum output occurred in 2011 (*n* = 121, 6.38%). In terms of total citations, there was a peak in 2014 (*n* = 2,899) (Figure 2A), and 2013 (*n* = 163, GR = 14.79%), 2014 (*n* = 194, GR = 19.02%), 2016 (*n* = 218, GR = 11.22%), and 2019 (*n* = 273, GR = 15.68%) were considered “remarkable” years (14) [note that a year is defined as remarkable if more than 150 papers were published and if there was a year-over-year growth rate (GR) >10% (Figure 2B)].

Pearson's correlation analysis showed that the annual output was positively correlated with the year (*r* = 0.983, *P* < 0.0001), and the linear fit (Figure 2C) between the year and the number of published MMA studies was significantly correlated (*R*^2^ = 0.9654, *P* < 0.0001). According to the mathematical model, publication output will reach 279 in 2020.

### Countries/Regions

**Figure 3:**
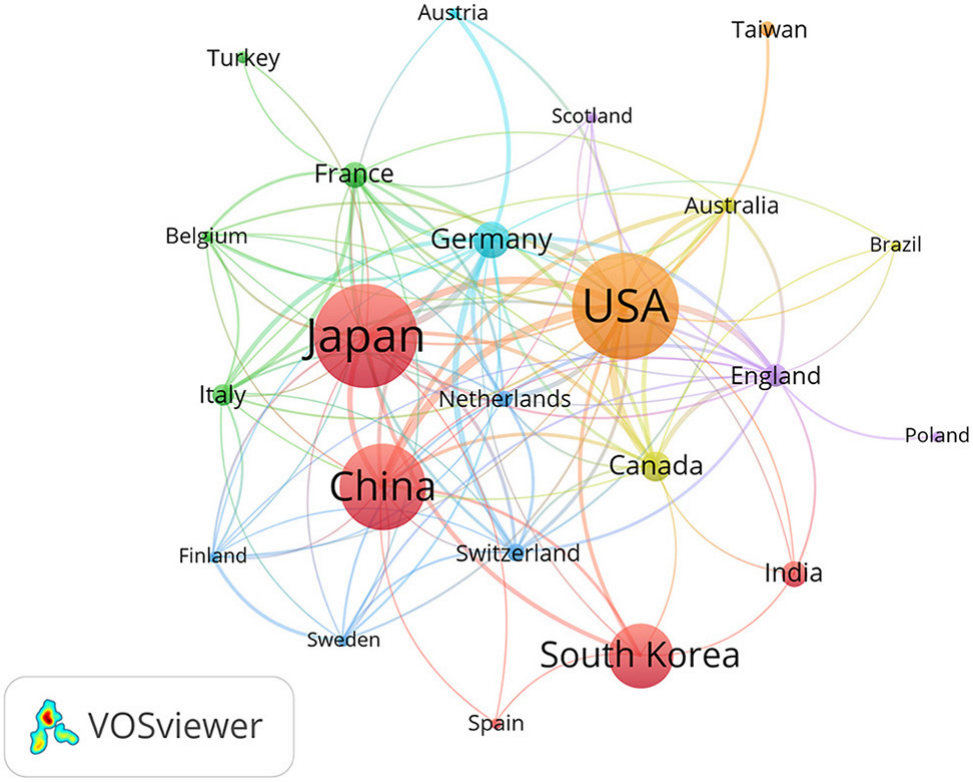
The co-authorship network visualization map of countries/regions related to MMA. Lines between clusters represented occurrence and the size of node was positively associated with the number of publications.

A total of 56 countries/regions contributed to the published MMA research (*n* = 1,896). The USA (*n* = 473, 24.95%) was the most prolific country/region. The top 10 productive countries/regions were shown in Table 1. A co-authorship network was constructed for the countries/regions using VOSviewer. The map of the co-authorship network (Figure 3) includes 23 countries, and the smallest node has 6 publications. The USA, Japan, China, and South Korea were represented by the four largest nodes, which is consistent with the result in Table 1. The USA had the strongest collaboration network, with the maximum total link strength (TLS = 127). The strongest collaboration networks were between the USA and China (TLS = 21) and between the USA and Canada (TLS = 21).

**Table 1:** The top 10 countries according to total publications during 2010–2019.

**Rank**	**Country**	**Number of publications**	**Proportion (%)**	**Total citations**	**Citations/** **paper**
1	The USA	473	24.95%	5,892	12.5 (7)
2	Japan	458	24.16%	5,906	12.9 (5)
3	China	347	18.30%	2,993	8.6 (8)
4	South Korea	231	12.18%	3,205	13.9 (3)
5	Germany	102	5.38%	1,304	12.8 (6)
6	Canada	72	3.80%	1,308	18.2 (1)
7	India	62	3.27%	191	3.1 (10)
8	France	59	3.11%	766	13 (4)
9	England	51	2.69%	745	14.6 (2)
10	Italy	47	2.48%	341	7.3 (9)

*Data were retrieved from 1,896 publications with VOSviewer on August 14, 2020*.

### Institutions

**Figure 4:**
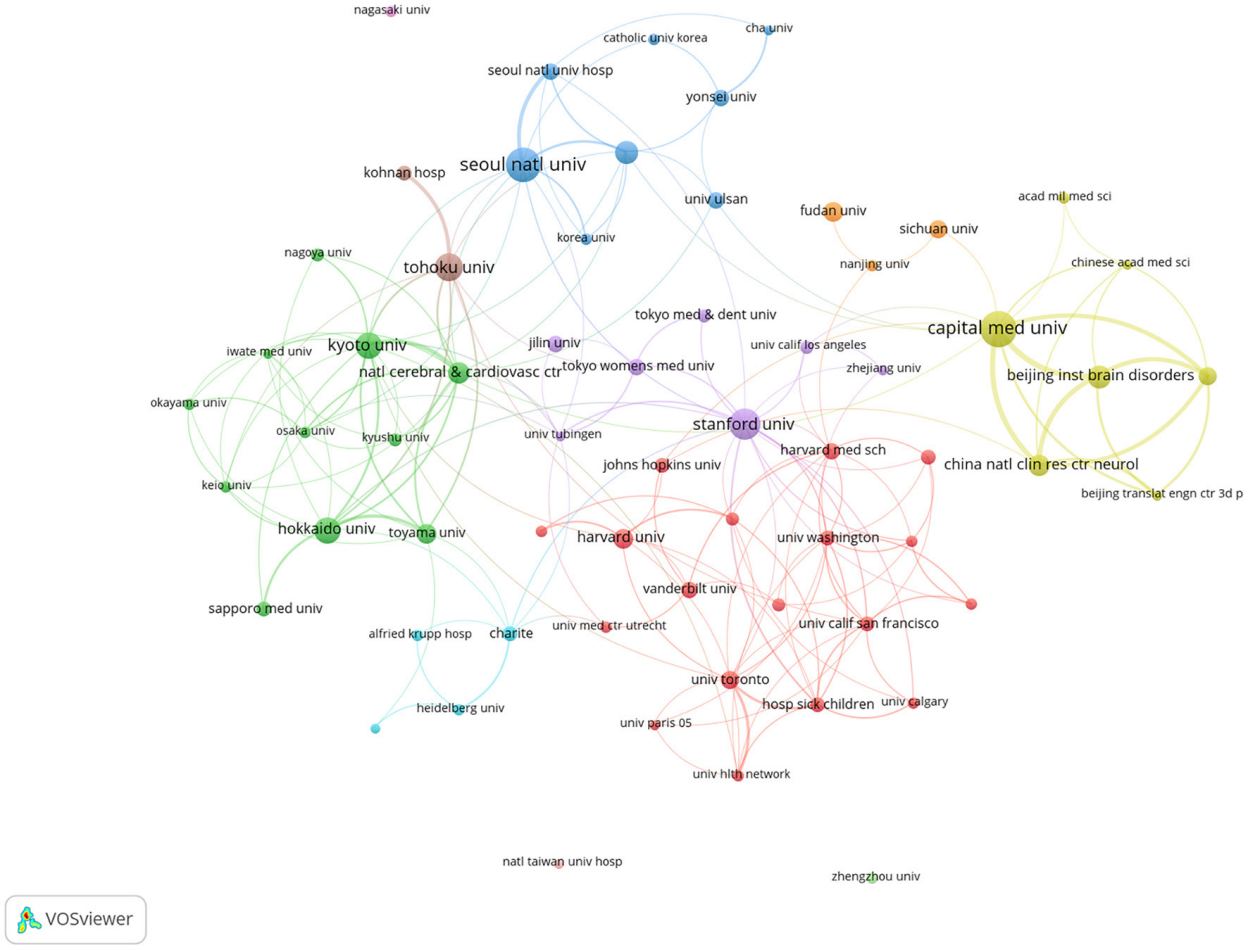
The co-authorship network visualization map of institutions for MMA research.

The top 10 institutions in terms of productivity were presented in Table 2. Among these institutions, Capital Medical University (China, 95 publications) was the most prolific institution, followed by Seoul National University (South Korea, 84 publications) and Stanford University (the USA, 71 publications). In terms of citations, Tohoku University (Japan, 1,633 times), Seoul National University (South Korea, 1,453 times), Kyoto University (Japan, 1,408 times) and Stanford University (the USA, 1,300 times) exceeded 1,000 citations. Cooperative relationship among 63 institutions is shown in Figure 4. The co-authorship institutional analysis network had a minimum threshold of 10 publications. As shown in the network map, a variety of institutions closely cooperation with each other.

**Table 2:** The top 10 most productive institutions between 2010 and 2019.

	**2010–2019**			
**Rank**	**The name of institution**	**Publications**	**Citations**	**Location**
1	Capital Med Univ	95	661	China
2	Seoul Natl Univ	84	1,453	Korea
3	Stanford Univ	71	1,300	The USA
4	Tohoku Univ	63	1,633	Japan
5	Kyoto Univ	57	1,408	Japan
6	Hokkaido Univ	55	821	Japan
7	Beijing Inst Brain Disorders	45	221	China
8	Sungkyunkwan Univ	41	703	Korea
9	Natl Cerebral & Cardiovasc Ctr	39	994	Japan
10	China Natl Clin Res Ctr Neurol Dis	37	187	China

*Data were retrieved from 1,896 publications with VOSviewer on August 14, 2020*.

### Journals and Co-cited Journals

Peer-reviewed journals publishing literature on MMA were identified with VOSviewer (*n* = 384). The top 10 journals and co-cited journals were shown in Table 3. The top 10 journals in terms of productivity collectively produced 714 publications, accounting for 37.7% of all papers, and STROKE had the highest impact factor (IF = 7.19). World Neurosurgery was the most prolific journal, with 168 publications. The top 3 co-cited journals were as follows: STROKE (5,721 co-citations), *Journal of Neurosurgery* (3,289 co-citations) and *Neurosurgery* (2,410 co-citations).

**Table 3:** The top 10 journals and co-cited journals on MMA research between 2010 and 2019.

**Rank**	**Journal**	**Publication number**	**Citation**	**IF^#^**	**Co-cited journal**	**Co-citation**	**IF**
1	*World Neurosurgery*	168	796	1.829	*Stroke*	5,721	7.19
2	*Journal of Stroke and Cerebrovascular Diseases*	84	555	1.787	*Journal of Neurosurgery*	3,289	3.968
3	*Journal of Neurosurgery*	80	1,108	3.968	*Neurosurgery*	2,410	4.853
4	*Stroke*	74	2,080	7.19	World Neurosurgery^*^	1,585	1.829
5	*Journal of Neurosurgery-Pediatrics*	58	575	2.117	*American Journal of Neuroradiology*	1,504	3.381
6	*Childs Nervous System*	56	323	1.298	*Clinical Neurology and Neurosurgery*	1,415	1.53
7	*Neurologia medico-chirurgica*	54	704	1.836	*Neurology*	1,293	8.77
8	*Acta Neurochirurgica*	48	522	1.817	*New England Journal of Medicine*	1,024	74.699
9	*American Journal of Neuroradiology*	47	708	3.381	*Acta Neurochirurgica*	986	1.817
10	*Neurosurgery*	45	844	4.853	*Cerebrovascular Diseases*	948	2.698

*Data were retrieved from 1,896 publications with VOSviewer on August 14, 2020*.

* *(Surgical Neurology was renamed World Neurosurgery in 2010)*.

# *Abbreviation for Impact Factor*.

### Authors and Co-cited Authors

A total of 6,744 authors contributed to MMA-related research, with an average of 3.6 authors per study. The top 12 contributing authors involved in the research were listed in Table 4. Zhang D was the most prolific author, with 61 publications, followed by Tominaga T (*n* = 59) and Fujimura M (*n* = 52). Co-cited authors were authors who have been co-cited in publications, and co-citation is a key measurement of the contribution degree of an author. The top 12 co-cited authors were shown in Table 4.

**Table 4:** The top 12 prolific authors and co-cited authors on MMA research from 2010 to 2019.

	**Author**				**Co-cited authors**		
**Rank**	**Name**	**Publications**	**Citations**	**Country**	**Name**	**Co-citations**	**Country**
1	Zhang D	61	474	China	Kuroda S	878	Japan
2	Fujimura M	52	1,140	Japan	Suzuki J	854	Japan
3	Tominaga T	50	1,268	Japan	Fujimura M	747	Japan
4	Wang R	49	459	China	Scott RM	704	The USA
5	Houkin K	45	753	Japan	Fukui M	449	Japan
6	Zhao JZ	44	397	China	Houkin K	416	Japan
7	Kuroda S	42	702	Japan	Miyamoto S	335	Japan
8	Miyamoto S	41	1,099	Japan	Liu WY	264	China
9	Zhang Y	40	254	China	Kim SK	233	South Korea
10	Steinberg GK	36	686	The USA	Matsushima T	211	Japan
11	Kim SK	36	575	South Korea	Karasawa J	206	Japan
12	Zhang Q	36	224	China	Matsushima Y	206	Japan

*Data were retrieved from 1,896 publications with VOSviewer on August 14, 2020*.

**Figure 5:**
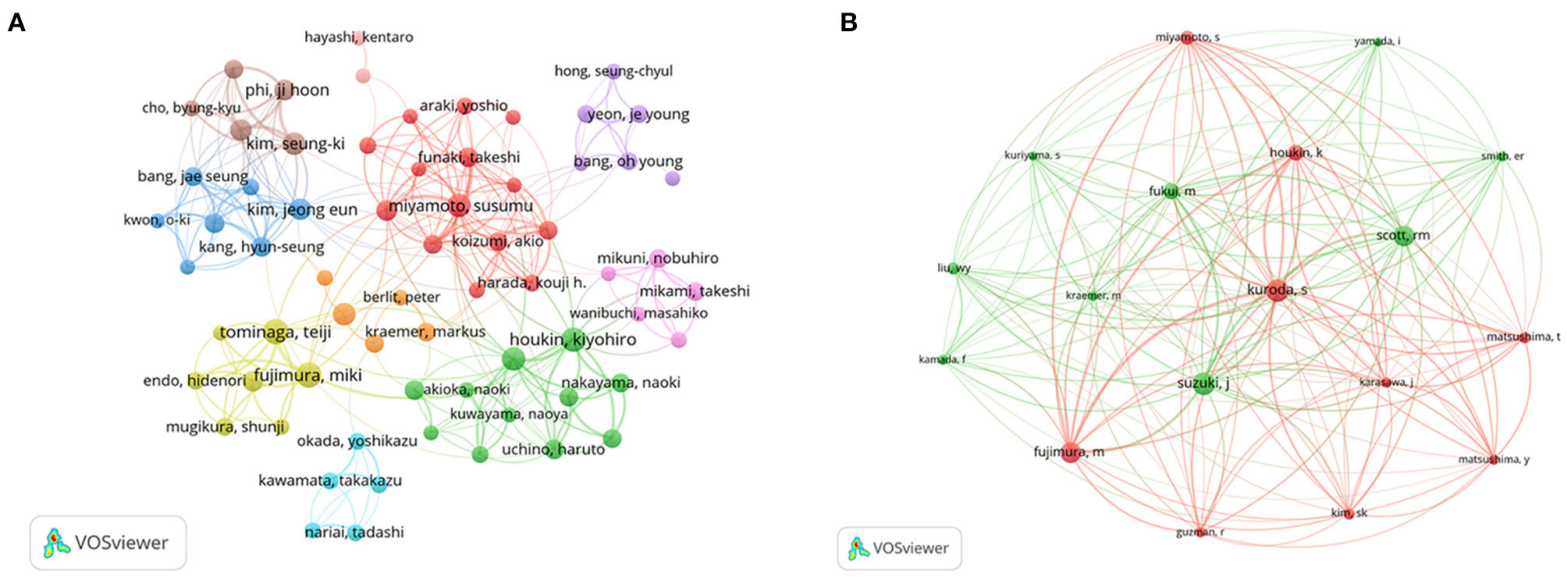
**(A)** Co-authorship network visualization map of authors on MMA research. **(B)** Co-citation network visualization map of authors on MMA research.

Visual maps of authors and co-cited authors can effectively display powerful research teams and potential partners (15). A co-authorship analysis of authors was carried out by VOSviewer. The minimum number of publications of each author was set to 10, and 106 authors were screened. Among them, some authors were not connected to others. To improve the visualization, the largest subnetwork (67 authors) was identified, as illustrated in Figure 5A. Figure 5B displays the network map of co-cited authors with more than 230 co-citations. In the map, Kuroda S was the most notable author in terms of co-citations.

### Subject Categories

**Figure 6:**
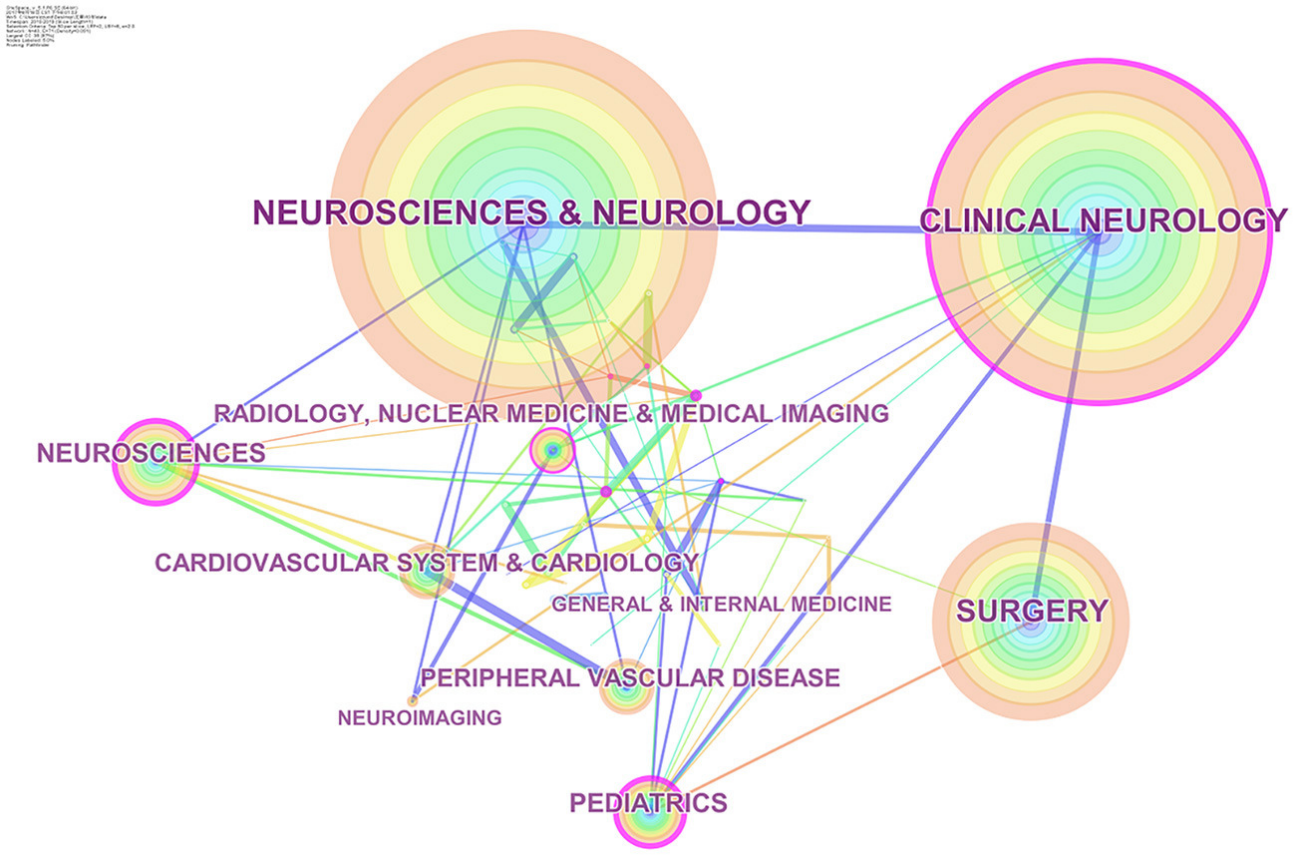
The visualization map of subject categories on MMA. The tree ring-shaped nodes represented different subject categories. The lines between two nodes meant co-occurrence. The area of the nodes referred to the number of publications. Nodes with high centrality (purple ring) were deemed as the hot field.

The map of publication categories (Figure 6) was generated using CiteSpace software. Centrality indicates the importance of an item in CiteSpace. All 1,896 publications were associated with 40 disciplines. The top 10 subject categories ranked by publication or centrality were shown in Table 5, and NEUROSCIENCES AND NEUROLOGY and BIOCHEMISTRY AND MOLECULAR BIOLOGY tied for first.

**Table 5:** Top 10 subject categories in terms of publication number and centrality related to MMA research.

**Rank**	**Publications**	**Category**	**Centrality**	**Category**
1	1,364	NEUROSCIENCES AND NEUROLOGY	0.37	BIOCHEMISTRY AND MOLECULAR BIOLOGY
2	1,188	CLINICAL NEUROLOGY	0.35	PEDIATRICS
3	710	SURGERY	0.25	CLINICAL NEUROLOGY
4	302	NEUROSCIENCES	0.23	BIOPHYSICS
5	256	PEDIATRICS	0.21	NEUROSCIENCES
6	237	CARDIOVASCULAR SYSTEM AND CARDIOLOGY	0.19	RADIOLOGY, NUCLEAR MEDICINE & MEDICAL IMAGING
7	224	PERIPHERAL VASCULAR DISEASE	0.18	HEMATOLOGY
8	184	RADIOLOGY, NUCLEAR MEDICINE AND MEDICAL IMAGING	0.18	CELL BIOLOGY
9	83	NEUROIMAGING	0.13	CARDIAC AND CARDIOVASCULAR SYSTEMS
10	74	GENERAL AND INTERNAL MEDICINE	0.09	CARDIOVASCULAR SYSTEM AND CARDIOLOGYGENETICS AND HEREDITYONCOLOGY

*Data were retrieved from 1,896 publications with CiteSpaceV on August 14, 2020*.

### Keywords

**Figure 7:**
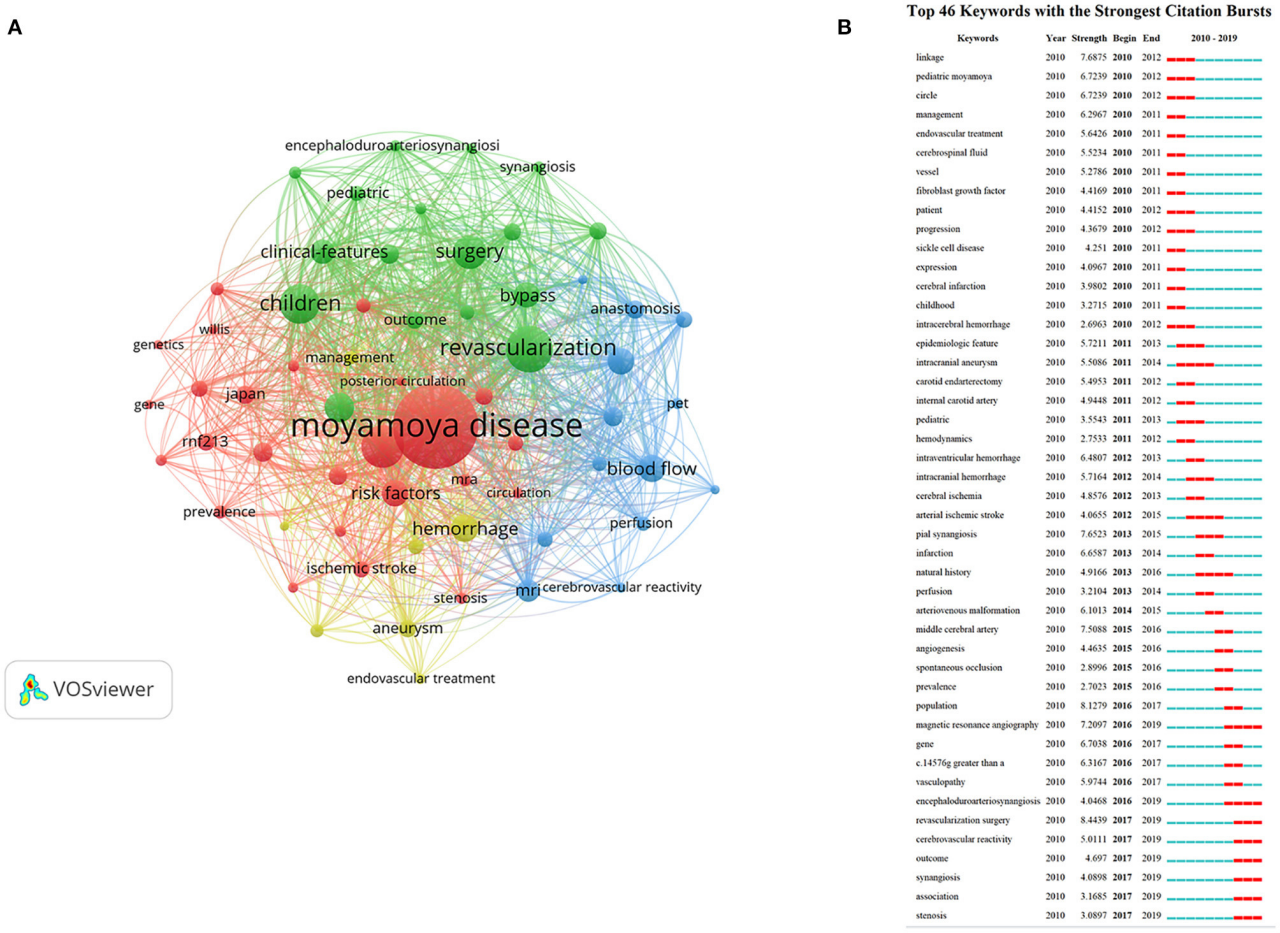
**(A)** The co-occurrence network visualization map of keywords in the MMA research field from 2010 to 2019. Keywords in the same color represent were sorted into the same cluster. **(B)** The top 46 keywords with the strongest citation bursts on MMA research between 2010 and 2019. The red segment of the blue line denoted the burst duration of a keyword.

High-frequency keywords could reflect research hot spots. A thesaurus was used to clean the data, and 4,619 keywords were extracted from the 1,896 publications. Ultimately, 62 keywords with more than 30 occurrences were identified. The top 5 keywords ranked by number of occurrences were as following: moyamoya disease (*n* = 1,081), revascularization (*n* = 421), stroke (*n* = 372), children (*n* = 331), and surgery (*n* = 258). The co-occurrence network of keywords is displayed in Figure 7A. As shown in Figure 7A, the keywords were grouped into four clusters. Notably, the primary keywords for cluster 1 (red) referred to epidemiology and genetics and included epidemiologic features, Japan, prevalence, gene, genetics, and RNF213. Cluster 2 (green) referred to surgical treatments and included revascularization, surgery, pial synangiosis, encephaloduroarteriosynangiosis, synangiosis, and indirect revascularization. Cluster 3 (blue) referred to imaging diagnosis and included blood flow, MRI, hemodynamics, perfusion, PET, cerebrovascular reactivity, and SPECT, and cluster 4 (yellow) referred to clinical presentation and prognosis and comprised hemorrhage, aneurysm, subarachnoid hemorrhage, and natural history.

Burst terms were identified with CiteSpace to indicate new research trends and frontier topics (16). The node type was set as “Keyword,” and other parameters were set in accordance with the description in the Materials and Methods section. The minimum duration was set to the default value of 2. As Figure 7B shows, 46 keywords with strong citation bursts were found. Among the top keywords, revascularization surgery had the highest burst strength (8.4439). Other keywords with high burst strengths from 2017 to 2019 include revascularization surgery, cerebral vascular reactivity, outcome, synangiosis, association, and stenosis.

### Co-cited References

**Figure 8:**
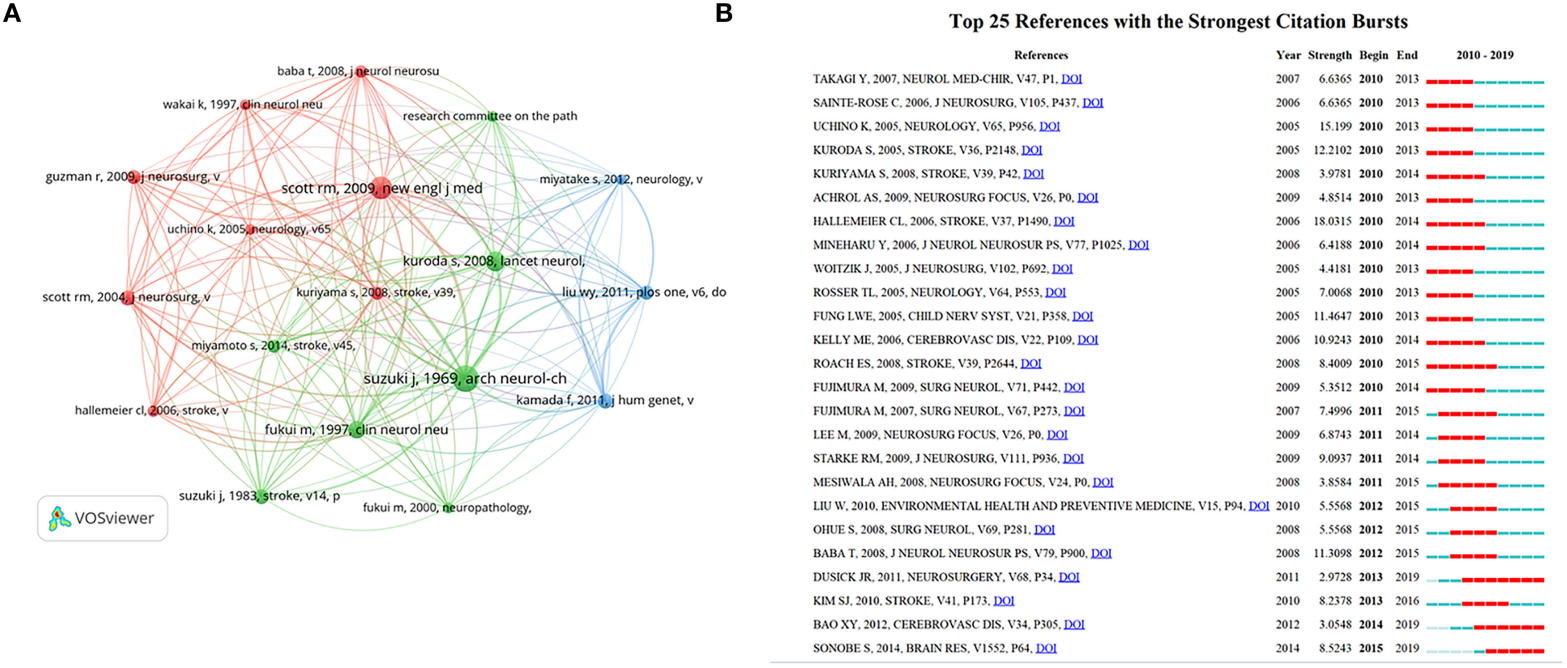
**(A)** The Co-citation network visualization map of references on MMA research between 2010 and 2019. **(B)** The top 25 references with the strongest citation bursts on MMA research between 2010 and 2019. The red segment of the blue line denoted the burst duration of a keyword.

Co-cited references were references cited collectively in the reference lists of other literature (17). Among the 1896 publications, 24,041 co-cited references were identified. The top ten co-cited references were presented in Table 6. The article published in *Arch Neurol-Chicago* (18) written by Suzuki J and colleagues was the most co-cited reference (*n* = 615), followed by the review written by Scott RM in The New England Journal of Medicine (*n* = 452) (2) and the review written by Kuroda S in *Lancet Neurology* (*n* = 354) (1). References with more than 100 co-citations were used to form a co-citation network map. In Figure 8A, the study published in *Arch Neurol-Chicago* (18) has the highest weight and an active relationship with other studies.

**Table 6:** The top 10 co-cited reference with regard to MMA during 2010–2019.

**Rank**	**Co-cited reference**	**Count**
1	Suzuki J, 1969, *Arch Neurol-Chicago*, V20, P288 (18)	615
2	Scott RM, 2009, *New Engl J Med*, V360, P1226 (2)	452
3	Kuroda S, 2008, *Lancet Neurol*, V7, P1056 (1)	354
4	Fukui M, 1997, *Clin Neurol Neurosur*, V99, PS238 (19)	269
5	Scott RM, 2004, *J Neurosurg*, V100, P142 (20)	204
6	Kamada F, 2011, *J Hum Genet*, V56, P34 (21)	193
7	Suzuki J, 1983, *Stroke*, V14, P104 (22)	190
8	Liu WY, 2011, *PLoS ONE*, V6 (23)	188
9	Guzman R, 2009, *J Neurosurg*, V111, P927 (24)	187
10	Kuriyama S, 2008, *Stroke*, V39, P42 (25)	166

*Data were retrieved from 1,896 publications with VOSviewer on August 14, 2020*.

Citation bursts can be analyzed by identifying references that researchers focused on during certain periods of time (26). References with the strongest citation bursts were identified using CiteSpaceV, and the minimum burst duration was confined to 4 years. The node type was set as “Cited Reference,” and the other parameters were set in accordance with the description in the Materials and Methods section. As shown in Figure 8B, burst strength values of the top 25 references with the strongest citation bursts ranged from 2.9728 to 18.0315. “Hallemeier CL, 2006, *Stroke*, V37, P1490 (27)” had the highest burst strength (18.0315), and three co-cited references had recent bursts: “Dusick JR, 2011, *Neurosurgery*, V68, P34 (28),” “Bao XY, 2012, *Cerebrovasc Dis*, V34, P305 (29),” and “Sonobe S, 2014, *Brain Res*, V1552, P64 (30).” Significantly, the third article by Sonobe S showed the highest burst strength (8.5243) among studies with citation bursts ending in 2019. Dusick JR demonstrated that indirect revascularization surgeries by encephaloduroarteriosynangiosis (EDAS) and multiple burr-hole operation provided effective prevention for recurrent ischemia and hemorrhage in 95% children and adults (28). Bao XY revealed EDAS in Chinese adult patients with MMD was relatively safe and effective at preventing future ischemia and improving quality of life (29). SONOBE S and colleagues found that the functional deficiency of RNF213 did not sufficiently cause the emergence of the MMD-like phenotype (30).

Revascularization surgery researches and RNF213-related researches were selected as recent frontier topics. “Revascularization surgery” had the highest burst strength among the six recent keywords. It was also described in the first article with citation bursts ending in 2019 by Dusick JR (28). “RNF213” was discussed in the third article by Sonobe S (30), which also represented the hot spots of MMA.

## Discussion

Although MMA is a rare cerebrovascular disease, it is one of the main causes of pediatric and young adult stroke (31, 32). The etiology and pathogenesis mechanisms of MMA remained unclear, and there is currently a lack of effective medical treatments. Surgical intervention is currently the main therapeutic strategy. However, surgical treatment mainly aims to improve cerebral hypoperfusion rather than combat the pathogenic mechanisms (33). Timely diagnosis and early intervention have benefitted MMA patients (34), but it is still necessary to establish a sufficient understanding of this disease. Therefore, we made a novel attempt to examine MMA publications from 2010 to 2019 and to provide a full view of the research trends by using a bibliometric analysis.

The data of annual output and growth rate could reflect the development of a given research area in terms of scientific data. In 2011, RNF213 was reported as the first susceptibility gene by Kamada F et al. and Liu WY et al. respectively (21, 23). This discovery advanced the study of genetic factors in MMD pathogenesis. More RNF213-related articles were published after 2011. These two articles were also shown in Table 6. This may be the reason why the growth rate rapidly increased in 2011–2012. And in 2012, Japan released new guidelines for diagnosis and treatment of MMD (35). This guideline was recognized worldwide and drove research discovery in related area.

Japan, China, and South Korea belong to the same cluster, as shown in Figure 3, and they were ranked the highest in Table 1. Furthermore, nine of the top 10 productive institutions were in East Asian countries, which shows that East Asian institutions occupied key positions in MMA research, which is consistent with the epidemiological understanding that MMA has a higher incidence rate in East Asia (36).

As shown in Table 3, World Neurosurgery had the largest number of publications, and STROKE had the most co-cited article. An understanding of prolific journals could help researchers choose journals for draft submissions, and publications from top co-cited journals could be used as authoritative references. Moreover, there were six journals that were in both the top 10 prolific journals and the top 10 co-cited journals: *World Neurosurgery, Journal of Neurosurgery, Stroke, Acata Neurochirurgica, American Journal of Neuroradiology*, and *Neurosurgery*. All six journals were strongly recommended by researchers in the field.

In terms of subjects in Figure 6 and Table 5, NEUROSCIENCES AND NEUROLOGY and BIOCHEMISTRY AND MOLECULAR BIOLOGY occupied pivotal positions in this field, indicating that both clinical practice and mechanism-related studies were of vital importance for MMA research.

Generally, a co-cited reference that ranked higher represented an “intellectual base” in this area (9, 37). These publications could provide a foundation for scholars who want to acquire quick insight in a particular field.

As shown in Result section, revascularization surgery and RNF213 were chosen as recent frontier topics in our study.

(1) Revascularization surgery played a significant role in the treatment of MMA patients. Surgical revascularization surgeries could be divided into direct revascularization, indirect revascularization, and the combination of both (38). As for adult patients with MMD, direct revascularization and combined revascularization were recommended (39, 40). Indirect revascularization was feasible for certain patient subgroups such as pediatric patients as well (41). The Japanese MMD trial (JAM) by Miyamoto S, et al. provided the highest-level evidence of the preventive effect of direct anastomosis for hemorrhagic MMD (42). Remarkably, the paper appeared in Figure 8A.

There were still some lingering questions remained to be elucidated. Although revascularization surgery might well be the most effective method for ischemic MMD in clinical practice, randomized clinical trials were still lacking (43, 44). The small sample size and lacking of neurological function assessment restrained the clinical applications of the JAM trial. Besides, ischemic events, especially transient ischemic attacks, were the main clinical manifestation for children and hemorrhagic stroke was more generally seen in adults (45–47). The occurrence rate of hemorrhagic stroke in adults varied among different region (47). Furthermore, cognition preservation was also of great importance. Revascularization surgery has been proved to be beneficial to patients with concomitant cognitive impairment (48), while a recent study revealed that cerebral hyperperfusion in the acute phase after revascularization surgery could result in cognitive impairment (49). Comprehensive evaluation and precise diagnosis were desperately needed to provide reliable basis for surgery opportunity choice and clinical treatments. Hence, large scale multicenter and multination RCTs were needed to illustrate the role of revascularization surgery among various populations of different epidemiological backgrounds. However, it was noteworthy that RCTs for ischemic MMD were ethically difficult to be carried out. Ongoing trials, such as the Adult Hemorrhagic Moyamoya Surgery Study (AHMMS), might provide insights to some of these questions (50). The aim of the AHMSS study was to replicate the therapeutic efficacy for preventing rebleeding in Chinese adult patients with hemorrhagic MMD and to reveal whether extracranial–intracranial (EC–IC) bypass surgery could improve neurological function, which was not evaluated in the JAM trial.

(2) MMA has been reported to be related to genetic factors, and numerous studies have revealed that the RNF213 gene on chromosome 17q25.3 played a role in the pathogenesis of MMA (51). Screening for RNF213 gene susceptibility to MMA patients and their families might provide evidence for the early diagnosis of MMA disease (52). A variant in RNF213 that altered arginine at position 4810 (p.R4810K) was associated with MMD in Asian populations. The homozygote of the p.R4810K variant on RNF213 exhibited an early onset age and severe form of moyamoya disease (53). Alterations in RNF213 predisposed patients of diverse ethnicities to MMD, but the p.R4810K variant predisposed individuals of Asian populations only (54). R4810K was an AAA(+) ATPase and decreased ATPase activity, suggesting its antiangiogenic activity through stabilizing oligomers. Therefore, a specific inhibitor of ATP binding to the first AAA(+) could be a promising therapeutic candidate for MMD (55).

This article also had some limitations:

1) Publication bias: To match the data type requirements of scientometric tools such as CiteSpace and VOSviewer, we extracted data from WoSCC, one of the most extensive and comprehensive global databases and the most commonly used source of publications in scientometry. Articles not indexed in WoSCC could not be involved in our study. As a result, the bias is hard to be avoided objectively.
2) Language bias: Identified literature were primarily published in English (ratio = 0.9879) which might lead to a language bias. English was not the main language used in East Asia, where MMA had a high incidence rate. MMA research written in Chinese, Japanese, and Korean had high reference value.
3) The larger number of publications was not always more important or informative. Researchers were recommended to pay attention to both the quantity of publications and their citation when they followed certain authors or institutions.

Bibliometric analysis could be used as a synopsis to help researchers to gain initial and general insights into a specific field. With the expansion of researchers' exploration of MMA, the hot spots of research on MMA have gradually changed. The brief contrasts between bibliometric data in 2000–2009 and 2010–2019 have been presented in Supplementary Material. Despite certain limitations, our study provided a comprehensive assessment and a preliminary understanding of the trends in MMA research. We sincerely hope that bibliometric and visualization-based analyses of global literature can provide an in-depth view of the disease mechanisms, epidemiology and treatments.

### Prospects

To our knowledge, this article presents the first bibliometric study that systematically analyses the global trends in MMA research over the past 10 years. This analysis could guide scholars in the selection of new research directions and help them to understand research hot spots and frontiers. Hopefully, high-quality clinical evidence will be obtained in the future. Further cooperation between authors, institutions and countries is expected to accelerate the treatment of MMA.

## Data Availability Statement

The original contributions presented in the study are included in the article/Supplementary Material, further inquiries can be directed to the corresponding author/s.

## Author Contributions

DC and GZ: study conception, design and data analysis. DC: paper writing. JiaW, SC, JinW, HN, and ZT: language polishing, paper review and editing. All authors read and approved the final version of the paper.

## Conflict of Interest

The authors declare that the research was conducted in the absence of any commercial or financial relationships that could be construed as a potential conflict of interest.
