# Supplementary material for: Mapping Trends in Moyamoya Angiopathy Research: A 10-Year Bibliometric and Visualization-Based Analyses of the Web of Science Core Collection (WoSCC)
Source: Front Neurol. 2021 Mar 2;12:637310. doi: 10.3389/fneur.2021.637310 (PMC7960774; doi:10.3389/fneur.2021.637310)
Supplement: Supplementary file 1 [file Data_Sheet_1.PDF]

Supplementary Material

Comparison of data between 2000-2009 and 2010-2019

A total of 787 studies were published in 232 journals by 2781 authors, 681 institutions and 48 countries/regions from 2000 to 2009. The number of publications has more than doubled from the first decade to the second decade of the 21st century. Compared with the first decade, more researchers, institutions and countries were involved in the field of MMA during 2010 to 2019. Significantly, Japan (n=268) was the most prolific country, followed by USA (n=186) and South Korea (n=80). China ranked sixteenth with 17 publications. "CLINICAL NEUROLOGY" ranked first in terms of the centrality, which indicated the importance of the item. The top ten co-cited references were presented in Table S1 and the top 21 references with the strongest citation bursts were shown in Figure S1. Notably, three top co-cited references in 2000-2019 by Ikeda H, Yamauchi T and Inoue TK revealed that D6S441, 17q25 and 3p24.2-p26 might be linked to familial moyamoya disease in Japan(1-3).

"Bypass surgery" had the highest burst strength (4.5369) in the study of MMA during 2000 to 2009. "Bypass surgery" and "Revascularization surgery" were keywords with the highest burst strength in two periods respectively, which indicated that clinical treatments of MMA have always been the focus of attention in this field.

According to Table S1, publications during 2000-2019 mainly focused on the surgical treatment for MMA, and the genetic mechanisms underlying MMA in this period were confined to the gene mapping of susceptibility gene, dictated by various technical constraints. Along with the establishment and promotion of clinical guidelines(4), the explorations of the etiology and mechanism of MMA have gradually become a hot spot in recent ten years. This phenomenon was consistent with our conclusion that RNF213-related researches might represent cutting-edge topic in 2010-2019. RNF213 was identified as the first susceptibility gene in Japanese patients and RNF213-related mechanism researches were also carried out in animal experiments step by step in recent years(5-7) , demonstrating the process of

researchers' understanding of MMA from the elementary to the profound.

Interestingly, as for the five co-cited references with recent bursts in 2000-2009, the first one was the study design for the JAM trial. Results of the JAM trial in the second period have provided the highest-level of evidence for of the preventive effect of direct anastomosis for hemorrhagic MMD up to now.

REFERENCES

1. Ikeda H, Sasaki T, Yoshimoto T, Fukui M, Arinami T. Mapping of a familial moyamoya disease gene to chromosome 3p24.2-p26. *Am J Hum Genet* (1999) 64(2):533-7. doi: 10.1086/302243. PubMed PMID: 9973290; PubMed Central PMCID: PMCPMC1377762.
2. Yamauchi T, Tada M, Houkin K, Tanaka T, Nakamura Y, Kuroda S, et al. Linkage of familial moyamoya disease (spontaneous occlusion of the circle of Willis) to chromosome 17q25. *Stroke* (2000) 31(4):930-5. doi: 10.1161/01.str.31.4.930. PubMed PMID: 10754001.
3. Scott RM, Smith JL, Robertson RL, Madsen JR, Soriano SG, Rockoff MA. Long-term outcome in children with moyamoya syndrome after cranial revascularization by pial synangiosis. *J Neurosurg* (2004) 100(2 Suppl Pediatrics):142-9. doi: 10.3171/ped.2004.100.2.0142. PubMed PMID: 14758941.
4. Research Committee on the P, Treatment of Spontaneous Occlusion of the Circle of W, Health Labour Sciences Research Grant for Research on Measures for Infractable D. Guidelines for diagnosis and treatment of moyamoya disease (spontaneous occlusion of the circle of Willis). *Neurol Med Chir (Tokyo)* (2012) 52(5):245-66. doi: 10.2176/nmc.52.245. PubMed PMID: 22870528.
5. Kamada F, Aoki Y, Narisawa A, Abe Y, Komatsuzaki S, Kikuchi A, et al. A genome-wide association study identifies RNF213 as the first Moyamoya disease gene. *J Hum Genet* (2011) 56(1):34-40. doi: 10.1038/jhg.2010.132. PubMed PMID: 21048783.
6. Liu W, Morito D, Takashima S, Mineharu Y, Kobayashi H, Hitomi T, et al. Identification of RNF213 as a susceptibility gene for moyamoya disease and its possible role in vascular development. *PLoS One* (2011) 6(7):e22542. doi: 10.1371/journal.pone.0022542. PubMed PMID: 21799892; PubMed Central PMCID: PMCPMC3140517.
7. Sonobe S, Fujimura M, Niizuma K, Nishijima Y, Ito A, Shimizu H, et al. Temporal profile of the vascular anatomy evaluated by 9.4-T magnetic resonance angiography and histopathological analysis in mice lacking RNF213: a susceptibility gene for moyamoya disease. *Brain Res* (2014) 1552:64-71. doi: 10.1016/j.brainres.2014.01.011. PubMed PMID: 24440776.
8. Suzuki J, Takaku A. Cerebrovascular "moyamoya" disease. Disease showing abnormal net-like vessels in base of brain. *Arch Neurol* (1969) 20(3):288-99. doi: 10.1001/archneur.1969.00480090076012. PubMed PMID: 5775283.
9. Suzuki J, Kodama N. Moyamoya disease--a review. *Stroke* (1983) 14(1):104-9.

doi: 10.1161/01.str.14.1.104. PubMed PMID: 6823678.
10. Karasawa J, Kikuchi H, Furuse S, Kawamura J, Sakaki T. Treatment of moyamoya disease with STA-MCA anastomosis. *J Neurosurg* (1978) 49(5):679-88. doi: 10.3171/jns.1978.49.5.0679. PubMed PMID: 712390.
11. Matsushima T, Inoue T, Suzuki SO, Fujii K, Fukui M, Hasuo K. Surgical treatment of moyamoya disease in pediatric patients--comparison between the results of indirect and direct revascularization procedures. *Neurosurgery* (1992) 31(3):401-5. doi: 10.1227/00006123-199209000-00003. PubMed PMID: 1407421.
12. Fukui M. Current state of study on moyamoya disease in Japan. *Surg Neurol* (1997) 47(2):138-43. doi: 10.1016/s0090-3019(96)00358-8. PubMed PMID: 9040816.
13. Fukui M. Guidelines for the diagnosis and treatment of spontaneous occlusion of the circle of Willis ('moyamoya' disease). Research Committee on Spontaneous Occlusion of the Circle of Willis (Moyamoya Disease) of the Ministry of Health and Welfare, Japan. *Clin Neurol Neurosurg* (1997) 99 Suppl 2:S238-40. PubMed PMID: 9409446.
14. Inoue TK, Ikezaki K, Sasazuki T, Matsushima T, Fukui M. Linkage analysis of moyamoya disease on chromosome 6. *J Child Neurol* (2000) 15(3):179-82. doi: 10.1177/088307380001500307. PubMed PMID: 10757474.
15. Ishikawa T, Houkin K, Kamiyama H, Abe H. Effects of surgical revascularization on outcome of patients with pediatric moyamoya disease. *Stroke* (1997) 28(6):1170-3. doi: 10.1161/01.str.28.6.1170. PubMed PMID: 9183345.

Table S1. The top 10 co-cited reference with regard to MMA during 2000 -2009

Rank	Co-cited reference	Count
1	Suzuki J, 1969,ARCH NEUROL-CHICAGO, V20, P288(8)	226
2	Suzuki J, 1983, STROKE, V14, P104(9)	132
3	Karasawa J, 1978, J NEUROSURG, V49, P679(10)	66
4	Ikeda H, 1999, AM J HUM GENET, V64, P533(1)	64
5	Matsushima T, 1992, NEUROSURGERY, V31, P401(11)	60
6	Fukui M, 1997, SURG NEUROL, V47, P138(12)	57
7	Fukui M, 1997, CLIN NEUROL NEUROSUR, V99, PS238(13)	56
8	Yamauchi T, 2000, STROKE, V31, P930(2)	56
9	Inoue TK, 2000, J CHILD NEUROL, V15, P179(14)	51
10	Ishikawa T, 1997, STROKE, V28, P1170(15)	51

Top 21 References with the Strongest Citation Bursts

References	Year	Strength	Begin	End	2000 - 2009
FUKUI M, 1997, SURG NEUROL, V47, P138, DOI	1997	4.2125	**2000**	2005	
EZURA M, 1995, CHILD NERV SYST, V11, P591, DOI	1995	3.0714	**2000**	2003	
YOSHIMOTO T, 1996, STROKE, V27, P2160, DOI	1996	4.159	**2000**	2004	
YAMADA I, 1995, RADIOLOGY, V196, P211, DOI	1995	3.4145	**2000**	2003	
HOUKIN K, 1996, NEUROLOGIA MEDICO-CHIRURGICA, V36, P783, DOI	1996	2.5327	**2000**	2004	
YAMADA I, 1995, RADIOLOGY, V197, P239, DOI	1995	3.4145	**2000**	2003	
GOLBY AJ, 1999, NEUROSURGERY, V45, P50, DOI	1999	4.4171	**2000**	2003	
KURODA S, 1995, CHILD NERV SYST, V11, P584, DOI	1995	4.1017	**2000**	2003	
HOUKIN K, 1996, STROKE, V27, P1342, DOI	1996	4.6257	**2000**	2004	
ISHIKAWA T, 1997, STROKE, V28, P1170, DOI	1997	2.805	**2001**	2005	
IKEZAKI K, 1997, STROKE, V28, P2513, DOI	1997	3.2937	**2001**	2004	
CRAMER SC, 1996, STROKE, V27, P2131, DOI	1996	4.2206	**2001**	2004	
HASUO K, 1998, JMRI-J MAGN RESON IM, V8, P762, DOI	1998	2.6646	**2001**	2005	
NAKASHIMA H, 1997, CLIN NEUROL NEUROSUR, V99, PS156	1997	3.1118	**2001**	2005	
ADAMS RJ, 1998, NEW ENGL J MED, V339, P5, DOI	1998	2.4966	**2002**	2005	
IMAIZUMI T, 1998, PEDIATR NEUROL, V18, P321, DOI	1998	3.9742	**2002**	2006	
MIYAMOTO S, 2004, NEUROL MED-CHIR, V44, P218, DOI	2004	3.8531	**2005**	2009	
SCOTT RM, 2004, J NEUROSURG, V100, P142, DOI	2004	7.7246	**2005**	2009	
LANTHIER S, 2000, NEUROLOGY, V54, P371, DOI	2000	2.5722	**2005**	2009	
KIM SK, 2002, NEUROSURGERY, V50, P88, DOI	2002	2.7184	**2005**	2009	
HOUKIN K, 2000, ACTA NEUROCHIR, V142, P269, DOI	2000	3.8179	**2005**	2009	

Figure legends

Figure S1. The top 21 references with the strongest citation bursts on MMA research between 2000–2009. The red segment of the blue line denoted the burst duration of a keyword.
